# Pancreas Transplantation from Donors after Circulatory Death: an Irrational Reluctance?

**DOI:** 10.1007/s11892-019-1238-y

**Published:** 2019-11-18

**Authors:** M. Leemkuil, H. G. D. Leuvenink, R. A. Pol

**Affiliations:** Department of Surgery, University of Groningen, University Medical Center Groningen, P.O. Box 30 001, 9700 RB Groningen, The Netherlands

**Keywords:** DCD, Pancreas transplantation, Preservation, Machine perfusion

## Abstract

**Purpose of Review:**

Beta-cell replacement is the best therapeutic option for patients with type 1 diabetes. Because of donor scarcity, more extended criteria donors are used for transplantation. Donation after circulatory death donors (DCD) are not commonly used for pancreas transplantation, because of the supposed higher risk of complications. This review gives an overview on the pathophysiology, risk factors, and outcome in DCD transplantation and discusses different preservation methods.

**Recent Findings:**

Studies on outcomes of DCD pancreata show similar results compared with those of donation after brain death (DBD), when accumulation of other risk factors is avoided. Hypothermic machine perfusion is shown to be a safe method to improve graft viability in experimental settings.

**Summary:**

DCD should not be the sole reason to decline a pancreas for transplantation. Adequate donor selection and improved preservation techniques can lead to enhanced pancreas utilization and outcome.

## Introduction

In a select group of patients with type 1 diabetes mellitus (DM) with severe complications, beta-cell replacement by either pancreas or islets of Langerhans transplantation is the treatment of choice, leading to restoration of normoglycemia, reduction of long-term diabetes complications, and improved quality of life [1, 2]. Results of pancreas transplantation have improved in the last decades by optimization of surgical techniques and immunosuppressive regiments [3, 4]. Since pancreas transplantation is not a direct life-saving operation, strict donor selection criteria are used when accepting a pancreas [2, 5]. Despite a growing incidence in type 1 DM worldwide, pancreas transplant numbers in the USA and Eurotransplant region are decreasing, whereas numbers in the UK remain practically stable [6]. The main reasons for this are the lack of good-quality donor grafts and improvement in DM treatment, even though pancreas transplantation leads to more stable glycated hemoglobin (HbA1c) levels compared with strict insulin regimens [7], and long-term results of simultaneous pancreas-kidney (SPK) transplantation demonstrate a clear survival benefit as compared with patients who remain on the waiting list [8]. Forced by donor shortage, nowadays, more extended criteria donors (ECD) are used for transplantation, i.e., donation after brain death donation after brain death donors (DBD) of higher age and BMI, or donation after circulatory death donors (DCD). Most transplanted pancreata originate from DCD Class III (controlled) and less frequently from Class IV (uncontrolled controlled) donors. Maastricht Class I and II (uncontrolled) donors generally are not used for pancreas transplantation [9]. Clinicians are often reluctant to accept DCD for pancreas transplantation given the higher risk of graft pancreatitis and thrombosis, leading to potentially devastating complications [2]. The aim of this review is to give an overview on the pathophysiology, risk factors, and outcome in DCD transplantation, to provide tools in selecting suitable pancreas donors and to describe ways to optimize these organs by different preservation methods to safely increase the pancreas donor pool.

### Donor Selection

It has been generally assumed that the pancreas is much more vulnerable to injury than other abdominal organs [10•]. Therefore, strict donor selection criteria are used in pancreas transplantation, resulting in much higher discard rates of donor pancreata for transplantation when compared with other abdominal organs [5]. As a tool to assess suitable pancreas donors, the Pre-Procurement-Pancreas-Suitability-Score (P-PASS) was introduced in 2008 by the Eurotransplant Pancreas Advisory Committee. This scoring system was based on pancreas acceptance rate and includes nine donor parameters: age, body mass index (BMI), duration of intensive care unit (ICU) stay, cardiac arrest, serum sodium, amylase, lipase, and catecholamine dose. A range and point weight for each variable was defined based on clinical expertise and known literature, whereby the variables age and BMI were given twofold higher impact than the other variables. Retrospective analysis of more than 3000 reported pancreas donors identified a P-PASS of 17 as a significant cutoff point (*p* = 0.001) for pancreas acceptance: pancreata from donors with P-PASS > 17 were three times more likely to be discarded [11]. Subsequently, Eurotransplant recommends that all donors with a P-PASS < 17 have to be considered for pancreas donation [12]. A drawback of the P-PASS is a lack of data on patient and graft survival in the initial report, as it was only based on the pancreas acceptance rate. Also, DCD is not included in the scoring system, while nowadays there is a shift towards increasing numbers of DCD [13].

In 2010, the pancreas donor risk index (PDRI) was designed using data from the organ procurement and transplantation network (OPTN) with the aim to identify factors associated with pancreas graft survival after 1 year [14]. This index includes donor factors as well as transplant factors: donor gender, age, race (black/Asian), BMI, height, cause of death, serum creatinine, DCD status, preservation time, and type of transplantation. PDRI was derived from a large data set and provides an index for direct comparison of a potential donor with a “standard donor.” This model can help in the decision to accept a pancreas and to compare the results after transplantation.

Several studies have tried to compare and validate the scoring systems in retrospective analyses, usually resulting in conflicting outcomes. In a retrospective study using an Eurotransplant cohort from 2004 to 2014 investigating the predictive value of both indices on pancreas allocation, PDRI was proven more useful than P-PASS to predict pancreas acceptance. However, the authors suggest that potential pancreas donors should never be rejected exclusively based on a high PDRI score and it should be used as a tool to estimate outcome [13]. The authors also suggest that factors as recipient selection and experience with pancreas transplantation should be included in the consideration to accept a donor pancreas for transplantation.

P-PASS has been evaluated to predict pancreas graft survival in different countries. In a study from Poland, P-PASS was a significant risk factor for 1-year pancreas graft survival; patients with a functioning graft received pancreata from donors with lower P-PASS. A small, but significant, difference in P-PASS was seen; 15.7 versus 16.4 (*p* < 0.03) [15]. In another study, a significant association between P-PASS > 17 and graft failure was only shown within 1 month after transplantation (*p* = 0.025); at 1, 5, and 10 years, this association was no longer demonstrated [16]. In a study from the Netherlands, no predictive value of P-PASS could be demonstrated [17].

No predictive value of PDRI on 1- and 5-year graft survival was observed in two studies [15, 18]. In a large UK cohort, PDRI was significantly associated with 1-year graft survival in simultaneous pancreas-kidneys (SPK) recipients; however, the survival difference between the groups with the highest and lowest risk was only 7% at 5 years after transplantation. One-year graft survival was higher in SPK recipients (88%) compared with pancreas transplant alone (PTA) and pancreas after kidney (PAK) recipients (77%) when they received a pancreas from donors with elevated PDRI (1.57–2.21) [19]. PDRI was found to be a significant predictor of pancreas graft survival in a Dutch study; however, also good results could be achieved with grafts from high-PDRI donors [17]. We therefore conclude that the strict use of donor selection tools has limited clinical value and might even lead to refusal of potentially transplantable pancreata. The characteristics of P-PASS and PDRI are summarized in Table 1.

**Table 1:** Characteristics of P-PASS and PDRI

	Aim of the scoring system	Variables included in the model	Association with pancreas acceptance	Prediction of graft survival	Drawbacks
P-PASS	To assess suitable pancreas donors, education of involved professionals to increase pancreas transplant rates	Donor age, BMI, ICU stay, cardiac arrest, sodium, amylase, catecholamine use	Pancreata with P-PASS > 17 compared with < 17 are discarded three times more often (*p* < 0.001) [11] Weak prediction of organ acceptance (AUC 0.67) [13]	1-year GS associated with P-PASS (15.7 versus 16.4, *p* < 0.03) [15] PPAS > 17 associated with graft failure after 1 month (*p* = 0.025), no association at 1, 5, and 10 years [16] No predictive value of P-PASS on GS [17]	Median P-PASS of organ donors has increased to 19 [13] Shift towards more DCD while DCD is not included in P-PASS The model is based on pancreas acceptance and not outcome after transplantation
PDRI	To identify factors associated with increased pancreas graft failure, prediction of 1-year graft survival	Donor sex, age, race, BMI, height, cause of death, creatinine, DCD, SPK/PAK transplantation, preservation time	Stronger prediction of organ acceptance (AUC 0.79). PDRI is proven more useful than P-PASS to predict pancreas acceptance [13]	No predictive value on 1- and 5-year GS [15, 18] Significant association with 1-year GS in SPK (HR = 1.52, *p* = 0.009) [19] PDRI > 1.5 is associated with decreased GS (HR = 1.792, *p* = 0.018) [16] PDRI > 1.24 is associated with reduced GS in multivariate analysis (*p* = 0.002) [17]	Despite strong association of high PDRI donors with decreased GS, good results can be achieved with high risk grafts (PDRI > 1.24) [17] PDRI alone cannot be used as a strict criterion for pancreas acceptance

*BMI*, body mass index; *ICU*, intensive care unit; *AUC*, area under the curve; *GS*, graft survival; *DCD*, donation after circulatory death; *SPK*, simultaneous pancreas-kidney; *PAK*, pancreas after kidney

### Mechanism of Injury in DCD

In DCD, organs are subjected to a period of warm ischemia, which is thought to have a detrimental effect on organ quality. However, there is no consensus when warm ischemia actually commences and how long an organ can sustain warm ischemia before becoming irreversibly damaged. In the USA, warm ischemia is defined to start after withdrawal of life support therapy (WLST) and ends at the initiation of cold perfusion, while in most European countries, it is defined to start after asystole in the donor. An arterial pressure below 50 mmHg or oxygen saturation below 70% is now considered to be more physiologically relevant than asystole, leading to the increasingly accepted concept of functional warm ischemia [10•]. WLST is commonly performed at the ICU department, and after declaration of dead and the 5-min “no-touch” period, the donor is taken to the operation room (OR), where a midline laparotomy and cannulation of the aorta are performed. Warm ischemia ends at the start of the cold flush with preservation solution via the aorta. Warm ischemia leads to a quick depletion of intracellular energy sources, such as adenosine triphosphate (ATP), and accumulation of toxic metabolites [10•, 20]. In our study on human pancreas preservation, ATP concentration in DCD pancreata was significantly lower compared with DBD pancreata after a median period of 6-h static cold storage (SCS) [21]. In a canine study on segmental autotransplantation after different periods of warm ischemia (30, 60, 90, and 120 min) followed by 24 h of SCS in University of Wisconsin (UW) solution, a decline in pancreas viability after prolonged warm ischemia was reported [22]. Pancreas grafts were considered functional when normoglycemia for at least 5 days after transplantation was maintained or by positive evaluation using an intravenous glucose tolerance test 1 week after transplantation. The viability was correlated to the ATP concentrations observed: the tissue concentration of ATP at the end of the preservation period was predictive for post-transplant outcome. The authors demonstrated that pancreata subjected up to 60 min of warm ischemia followed by 24 h of SCS were still functioning after transplantation [22].

Several procurement protocols are used in order to shorten the length of warm ischemia. In some countries, it is allowed to perform premortem cannulation of the femoral vessels to enable the start of cold preservation directly after declaration of death. Heparin and vasodilatative drugs are also administered just before WLST in some centers [23, 24]. WLST might take place at the OR instead of the ICU department, resulting in shorter warm ischemia time. These preliminary preparations are unusual in the Eurotransplant region and the UK.

Although by definition the warm ischemic period ends when cold flush has started, biologically, the organ still suffers from lack of oxygen. Therefore, recently, donor organ extraction time is considered to be important as well. This period covers part of the cold ischemia time (CIT); it starts directly after the cold flush in the donor and ends when the organ is retrieved from the body and kept on ice. Earlier studies reported that prolonged kidney extraction time leads to an increase in delayed graft function (DGF) [25] and prolonged liver extraction time seems to have an independent effect on liver graft outcome after transplantation [26]. To which extent, whether the duration of pancreas extraction time has an effect on pancreas graft survival has yet to be determined. It is however evident that as all other organs, the pancreas temperature during explantation is far from the desired 4 °C. It has been demonstrated that the core pancreas temperature rises to 16.5 °C during procurement, after an initial decline to 6.8 °C just after the cold flush. In an experimental group, in which additional ice slush was added around the pancreas, the core pancreas temperature remained 9 °C. Results after islet isolation in both groups showed improved outcome in the experimental group regarding to islet equivalent (IEQ), viability, and response to glucose [27]. The effect of the extraction time or pancreatic temperature on the outcome of solid organ pancreas transplantations is currently unknown, but given the vulnerability of the organ and the impact on other organs, it is likely that extraction time is a relevant risk factor.

In 2015, recommendations from a European expert group concerning DCD pancreas transplantation have been published. Maastricht Class III and IV donors can be reasonably used for vascularized pancreas transplantation, if warm ischemia is limited with a maximum of 30 min. A rapid retrieval technique with perfusion of the abdominal organs should be performed via an aortic cannula. During procurement, ice slush should be added into the lesser sac to ensure topical cooling of the pancreas. Preservation should be performed by static cold storage and preservation time should be minimized.

### Outcome of DCD Pancreas Transplantation

Only a few countries worldwide have used DCD for vascularized pancreas transplantation: the USA, Canada, Australia, the UK, the Netherlands, Belgium, Sweden, and Japan. Subsequently, only a few studies reporting outcome after DCD transplantation have been published so far. In 2016, a meta-analysis on all comparative cohort studies reporting the outcome after DCD and DBD pancreas transplantation was published by our group [28••]. It was concluded that 1-year pancreas graft survival for SPK transplantation did not differ between DBD and DCD. Two of the included studies reported equal long-term results after DCD and DBD pancreas transplants (3- and 10-year patient and graft survival). DCD pancreas recipients were however more prone to develop thrombosis resulting in a higher reoperation rate. Interestingly, this did not lead to a lower patient or graft survival. Different definitions of warm ischemia time (WIT) were used, so no overall median WIT could be calculated. Despite varied lengths of WIT, even up to 110 min, all studies described excellent graft survival rates after 1 year. In 2017, a systematic review on DCD pancreas transplantation was published with equal results regarding to outcome after transplantation. In a subanalysis, WIT and thrombosis rate were compared in studies which used premortem preparations versus studies in which these were not performed. Early femoral cannulation significantly reduced warm ischemia time with approximately 10 min, which is, however, not yet directly associated with graft failure. DCD pancreata were proven to have a significantly higher rate of thrombosis than DBD pancreata. In a subgroup analysis, this was not shown for DCD pancreata procured from donors where premortem heparin administration was used [29••].

Recently, three papers on the outcome of DCD compared with DBD pancreas transplantation have been published. They all described single-center experiences including small number of patients (DCD groups 10–21 patients and DBD groups 68–596 patients) with a median follow up between 1 and 2.7 years. Two studies reported comparable WIT lengths (30 and 31 min) [30, 31] while in the third center, WLST was performed at the theater or nearby, leading to short WIT (data not given) [32]. Median donor age in DCD did not differ from DBD donors in two studies: 32 year [30] and 21 year [32], while in the third study, DCD donors were significantly younger than DBD donors: 27 vs. 43 years (*p* = 0.003) [31]. Excellent graft survival was reported in all studies: 100% after 1 year [31, 32] and even still 100% after 6 years [30]. None of the studies reported complete thrombosis leading to graft failure after DCD transplantation. Kopp and colleagues reported an equal PDRI score in both DBD and DCD pancreas donors, however, after eliminating the DCD factor, PDRI in this group was significantly lower: 0.97 versus 1.61 in DCD and DBD respectively. As indicated in this study, good results can be achieved by transplanting DCD pancreata, if careful donor selection is performed [31]. When it comes to risk factors and predicting outcome after pancreas transplantation, it appears that DCD seems to play a less important role than initially thought and could therefore be considered to be a justified source of donor pancreata.

### Pancreas Preservation

Nowadays, protocols for DCD pancreas preservation are quite similar to DBD protocols and are based on the principle of reducing cellular metabolism by lowering the temperature of the organ by SCS. With every 10 °C drop in temperature, cellular metabolism decreases two- to threefold, thereby leading to reduced oxygen and ATP use and reduction of ischemic injury [33]. However, at 4 °C, 10% of metabolism is still maintained, resulting in depletion of ATP levels in the absence of oxygen [34]. Together with the rapid decline of energy sources during the warm ischemic period in DCD organs, this period can further lead to a cellular “oxygen debt,” which results in the production of radical oxygen species and increased injury during reperfusion [20, 35].

Preservation solutions have been developed to counteract ischemic injury [36]. These act mostly by reducing cellular swelling and maintaining pH balance and in some solutions oxygen-free-radical scavengers and precursors for ATP are added. Studies have shown that preservation with either UW, Histidine-Tryptophan-Ketoglutarate (HTK), Celsior and Institute Georgez Lopez-1 (IGL-1) did not show superiority over another [37]. Currently, no studies have been performed that analyzed the effect of different preservation solutions on DCD pancreas transplants. Given the differences in injury during DBD and DCD procedures, it is likely to accept that different treatments for these organs might be necessary [36]. Multiple techniques have been developed and tested in order to reduce ischemia-reperfusion injury after transplantation. These techniques are most of all focused on maintaining cellular ATP by delivery of high oxygen concentrations to the tissue. The most explored techniques for pancreas preservation will be explained further.

#### Two-Layer Method

In 1988, the two-layer method (TLM) was developed by a Japanese group with the intention to improve pancreas viability during preservation for islet isolation. This technique involves the addition of perfluorocarbon (PFC) to the preservation solution, in order to combine the characteristics of the preservation solution and PFC together to prevent injury and supply oxygen during preservation. PFC is an inert solution with a high capacity for dissolving oxygen [34]. By addition of PFC to a preservation fluid, two layers are formed with PFC at the bottom and the preservation solution on top. The organ is placed in the solution which is presaturated with oxygen or continuously oxygenated during preservation. Oxygen is delivered to the pancreas by passive diffusion, with the intention to achieve ATP production in the presence of precursors in the preservation solution [36, 37]. Experimental studies in small animals showed promising results of TLM regarding to ATP levels when compared with SCS [38]. The ability of oxygen to penetrate deep in the tissue of large animals and humans is however questioned. In porcine pancreata preserved by the TLM, ATP levels were nearly undetectable and indistinguishable from those preserved by SCS alone [39]. In a large retrospective study from Sweden, the outcome of 200 islet isolations from human pancreata was analyzed, from which 103 pancreata were preserved by TLM and 97 pancreata by SCS. No differences with regard to islet yield, purity, or dynamic glucose stimulation after islet isolation was demonstrated. Islet post-transplant function in recipients was also equal in both groups. Subgroup analysis showed that TLM did not improve outcome either after prolonged CIT or in elderly donors (> 60 years of age) [40]. Recently, no studies on TLM of the pancreas have been published and as far as we know, TLM is not routinely used for pancreas preservation.

#### Persufflation

In organ persufflation, humidified and filtered oxygen is bubbled directly to an organ via its vasculature. Oxygen persufflation can either be performed anterograde (through the arteries) or retrograde (through the portal vein). The technique was first discovered coincidentally in 1902 when compressed oxygen instead of blood was perfused through a feline heart, leading to continuation of heart contractility [41]. In the last decades, the beneficial effects of this technique have been analyzed in experimental settings in the heart, kidneys, and livers. It has been shown that with this relatively simple technique, oxygen could be delivered to organs during cold storage, whereby ATP levels could be replenished and maintained with associated reduction of oxidative stress and lipid peroxidation [42–45]. Research concerning pancreas persufflation has been focused on pancreas preservation for islet transplantation. Scott et al. reported in their study on anterograde persufflation of DBD human and DCD porcine pancreata via the splenic artery and celiac trunk that oxygen was uniformly distributed throughout the organ and ATP concentration was restored [38]. Persufflation dramatically improved tissue health as shown by distended capillaries and significantly less autolysis and cell death when compared with TLM [39]. In a rat model, portal venous persufflation of the pancreas has shown to be superior to SCS and HMP regarding outcome of islet isolation [46]. In a recent study on human pancreata, a lowered expression of inflammatory genes in isolated islets after persufflation compared with SCS was reported. Preservation time could be extended with persufflation without additional loss of islet function or viability [47]. This technique is promising for pancreas preservation, as it allows the delivery of oxygen properly to the pancreatic tissue, without major complications. However, no research has yet been performed on its use in pancreas preservation for solid organ transplantation.

#### Machine Perfusion

Hypothermic machine perfusion (HMP) is a technique which enables continuous circulation of the microvasculature with cold preservation solution, removing toxic metabolites and supporting ATP synthesis by delivery of oxygen and adenosine [36]. HMP has been evaluated in donor organ preservation for different reasons; to improve the quality of marginal donor organs, to extend the preservation time, and to test graft viability. Previous clinical studies in kidneys showed especially beneficial effects of HMP on graft function after DCD or ECD kidney transplantation [48], and this technique is nowadays standard of care in some countries, among which is the Netherlands. In experimental and clinical studies on human DCD livers, excellent results were reported after oxygenated HMP with a restoration of ATP and improved hepatobiliary function [49] and a 100% graft survival 6 months after transplantation [20].

Early studies on HMP of canine pancreata concluded that pancreas storage time could be extended up to 24 h, while retaining viability [50] and function of isolated islets after autotransplantation in dogs [51]. Another study reported that tissue flow rate during HMP was a predictable index of pancreatic graft viability [52]. There has always been a hesitation to HMP of the pancreas, because it is a low-flow organ with a delicate vasculature and HMP is associated with edema and congestion with the risk of thrombosis [36, 53]. Because HMP of the pancreas is relatively complex and results of SCS have improved after development of dedicated perfusion solution, HMP fell out of favor. Lately, however, there is renewed interest in HMP of the pancreas, mainly because the lack of good-quality pancreas donors forces the search to improve the quality of marginal donor pancreata. During the last 15 years, few studies on HMP of the pancreas have been reported. Most of them involved animal pancreata and focused on pancreas preservation for islet isolation. Twenty-four hours of HMP of porcine pancreata led to moderate edema without loss of function of the isolated islets. The edema seemed to aid in enzymatic digestion, leading to a higher islet yield and purity of isolated islets compared with 24 h of SCS [54, 55]. In a split lobe porcine model, the results of islet transplantation in diabetic mice after 24-h HMP were analyzed. All mice receiving islets from HMP preserved pancreata showed recovery in diabetes and high viability as measured by oxygen consumption [56]. A protective effect on graft histopathology by 6-h HMP was reported in DCD porcine pancreata [57].

In the first study on human pancreata, four human donor pancreata were perfused for 4 h by HMP after 13 h of SCS, resulting in an adequate amount of islets that could be isolated with excellent in vitro viability [58]. In a recent study performed by our team, 20 human pancreata were included, from which 10 (5 DCD and 5 DBD) were subjected to 6-h HMP and 10 (5 DCD and 5 DBD) to 6-h of additional SCS after a median period of 6-h SCS. Oxygenated HMP with 25 mmHg was performed via the splenic and mesenteric artery separately. Uniform perfusion of the pancreata as shown by fluorescence microscopy was obtained and perfusion flow increased during the first minutes of HMP before it stabilized. ATP concentration increased significantly after HMP in both DCD and DBD organs with a respectively 6.8-fold and 2.6-fold increase. Also, ATP concentration of DCD pancreata was significantly lower at the start of HMP (8.4 in DCD and 48.2 μmol/g protein in DBD), which corrected after HMP to equal ATP concentrations (100.5 in DCD and 109.3 μmol/g protein in DBD). HMP did not induce cellular injury or edema. In two DCD pancreata, islet isolation was performed with good viability and in vitro function [21]. Another study demonstrated that 24 h of HMP with a perfusion pressure of 25 mmHg did not lead to macroscopic edema on human pancreata as conformed by histological analysis. After 12 h of HMP, insulin, glucagon, and somatostatin staining were normal [35]. This evidence suggests that HMP of the human pancreas is a safe preservation method regarding tissue injury and edema formation and replenishment of ATP can be achieved. However, the main limitation of these studies is the lack of a post-transplant evaluation reperfusion model in a normothermic environment.

Normothermic machine perfusion (NMP) can be used to improve organ quality during preservation and facilitate the administration of drugs, and it allows viability testing in a physiological environment prior to transplantation [59]. In experimental settings, it has been successfully used for viability testing in human donor livers [60] and kidneys [61]. Viability assessment of porcine pancreata was performed using NMP, to examine the effect of 5-h HMP compared with SCS alone after a prolonged period of 24-h CIT [53]. During HMP, perfusion flow was stable and the grafts experienced a weight gain of 15.3–27.6%. Perfusion flow indices (PFI) remained stable during normothermic reperfusion in the pancreata that were attributed to HMP, whereas the SCS-only pancreata showed declined PFI during reperfusion. In the second part of the study, three discarded human pancreata were perfused by HMP after prolonged period of CIT (up to 56 h). Stable PFI was shown with only mild weight gain (3.9% and 14.7%). Functional assessment during NMP demonstrated beta-cell viability in these grafts, even after prolonged period of SCS [53]. In a study on five discarded human pancreata, 1–2 h of NMP was performed for viability assessment after a median CIT of 13 h. One pancreas with a CIT of over 30 h was excluded from the analysis, given the macroscopically ischemic appearance of the duodenum and therefore poor comparability to the other organs according to the authors. In the included four pancreas grafts, stable mean arterial flow was maintained during NMP, and insulin secretion was observed in all pancreata although tissue edema did occur in all grafts [59]. In a study evaluating NMP as a preservation, 2-h NMP was performed in four DCD porcine pancreata after a short WIT (8.3 ± 6.6 min) and CIT (34 ± 7.8 min). All pancreata became moderately to severely edematous, congested, and hemorrhagic during NMP. Extensive hemolysis occurred and the pancreas looked grossly ischemic at the end of preservation [62]. As these studies give an insight in the possibility of NMP for assessing pancreas viability, the technique should be further optimized to avoid hemolysis and edema.

## Conclusion

Despite a growing incidence in type 1 DM, pancreas transplantation rates are declining worldwide. As beta-cell replacement is the best therapeutic option for these patients, better assessment of possible pancreas donors is needed to expand the donor pool without compromising the outcome. This overview shows that excellent results can be achieved with DCD pancreas transplantation, when other detrimental donor characteristics are kept to a minimum. Donor selection tools are developed in order to assess potential pancreas donors. PDRI did show to have a predictive value in outcome; however, conflicting cutoff points have been found in different studies. PDRI can be used as a tool to estimate the risk of a certain donor pancreas; nevertheless, other transplant and recipient factors should be kept in consideration. DCD should not be the sole reason to decline a pancreas for transplantation, though accumulation of risk factors in these donors should be avoided. Hypothermic oxygenated machine perfusion seems a promising preservation method for these organs, as it safely replenishes ATP concentrations.

## References

[1] White SA, Shaw JA, Sutherland DE (2009). Pancreas transplantation. Lancet.

[2] Mittal S, Gilbert J, Friend PJ (2017). Donors after circulatory death pancreas transplantation. Curr Opin Organ Transplant.

[3] Kopp WH, Verhagen MJ, Blok JJ, Huurman VA, de Fijter JW, de Koning EJ (2015). Thirty years of pancreas transplantation at Leiden University Medical Center: long-term follow-up in a large Eurotransplant center. Transplantation.

[4] Gruessner RW, Gruessner AC (2013). The current state of pancreas transplantation. Nat Rev Endocrinol.

[5] Maglione M, Ploeg RJ, Friend PJ (2013). Donor risk factors, retrieval technique, preservation and ischemia/reperfusion injury in pancreas transplantation. Curr Opin Organ Transplant.

[6] Benjamens S, Leemkuil M, Margreiter C, Huurman VA, Leuvenink HG, Pol RA (2019). A steady decline in pancreas transplantation rates. Pancreatology.

[7] Giorgakis E, Mathur AK, Chakkera HA, Reddy KS, Moss AA, Singer AL (2018). Solid pancreas transplant: pushing forward. World J Transplant.

[8] Gruessner RW, Sutherland DE, Gruessner AC (2004). Mortality assessment for pancreas transplants. Am J Transplant.

[9] Thuong M, Ruiz A, Evrard P, Kuiper M, Boffa C, Akhtar MZ, Neuberger J, Ploeg R (2016). New classification of donation after circulatory death donors definitions and terminology. Transpl Int.

[10] • Berney T, Boffa C, Augustine T, Badet L, de Koning E, Pratschke J, et al. Utilization of organs from donors after circulatory death for vascularized pancreas and islet of Langerhans transplantation: recommendations from an expert group. Transpl Int. 2015. **Summary on recent use of DCD donors for pancreas transplantation and guidelines provided by an expert group with the aim to increase DCD pancreas utilization**. 10.1111/tri.12681 26340064

[11] Vinkers MT, Rahmel AO, Slot MC, Smits JM, Schareck WD (2008). How to recognize a suitable pancreas donor: a Eurotransplant study of preprocurement factors. Transplant Proc.

[12] Boer de J. ET pancreas allocation system (EPAS). Eurotransplant manual 2015. 2015.

[13] Kopp WH, de Vries E, de Boer J, Putter H, Schareck W, Samuel U, Braat AE (2016). Donor risk indices in pancreas allocation in the Eurotransplant region. Transpl Int.

[14] Axelrod DA, Sung RS, Meyer KH, Wolfe RA, Kaufman DB (2010). Systematic evaluation of pancreas allograft quality, outcomes and geographic variation in utilization. Am J Transplant.

[15] Smigielska K, Skrzypek P, Czerwinski J, Michalak G, Durlik M, Grochowiecki T (2018). Usefulness of pancreas donor risk index and pre-procurement pancreas allocation suitability score: results of the Polish national study. Ann Transplant.

[16] Ayami MS, Grzella S, Kykalos S, Viebahn R, Schenker P (2018). Pancreas donor risk index but not pre-procurement pancreas allocation suitability score predicts pancreas graft survival: a cohort study from a large German pancreas transplantation center. Ann Transplant.

[17] Blok JJ, Kopp WH, Verhagen MJ, Schaapherder AF, de Fijter JW, Putter H, Ringers J, Braat AE (2016). The value of PDRI and P-PASS as predictors of outcome after pancreas transplantation in a large European pancreas transplantation center. Pancreas.

[18] Salamanca-Bustos JJ, Campos-Hernandez JP, Sanchez-Hidalgo JM, Arjona-Sanchez A, Sanchez-Gonzalez A, Arenas-Bonilla AJ (2016). Validation of the pancreatic donor risk index in simultaneous pancreas-kidney transplantation performed in Cordoba Hospital from 2000 to 2015. Transplant Proc.

[19] Mittal S, Lee FJ, Bradbury L, Collett D, Reddy S, Sinha S, Sharples E, Ploeg RJ, Friend PJ, Vaidya A (2015). Validation of the pancreas donor risk index for use in a UK population. Transpl Int.

[20] van Rijn R, Karimian N, Matton APM, Burlage LC, Westerkamp AC, van den Berg AP, de Kleine RHJ, de Boer MT, Lisman T, Porte RJ (2017). Dual hypothermic oxygenated machine perfusion in liver transplants donated after circulatory death. Br J Surg.

[21] Leemkuil M, Lier G, Engelse MA, Ploeg RJ, de Koning EJP, ‘t Hart NA (2018). Hypothermic oxygenated machine perfusion of the human donor pancreas. Transplant Direct.

[22] Yuan CH, Li GC, Zhang H, Cheng Y, Zhao N, Liu YF (2004). Evaluation of the viability and energy metabolism of canine pancreas graft subjected to significant warm ischemia damage during preservation by UW solution cold storage method. World J Gastroenterol.

[23] Fernandez LA, Di Carlo A, Odorico JS, Leverson GE, Shames BD, Becker YT (2005). Simultaneous pancreas-kidney transplantation from donation after cardiac death: successful long-term outcomes. Ann Surg.

[24] Bellingham JM, Santhanakrishnan C, Neidlinger N, Wai P, Kim J, Niederhaus S, Leverson GE, Fernandez LA, Foley DP, Mezrich JD, Odorico JS, Love RB, de Oliveira N, Sollinger HW, D’Alessandro AM (2011). Donation after cardiac death: a 29-year experience. Surgery.

[25] Osband AJ, James NT, Segev DL (2016). Extraction time of kidneys from deceased donors and impact on outcomes. Am J Transplant.

[26] Adelmann D, Roll GR, Kothari R, Syed S, Burdine LJ, Tavakol M, Niemann CU (2018). The impact of deceased donor liver extraction time on early allograft function in adult liver transplant recipients. Transplantation.

[27] Lakey JR, Kneteman NM, Rajotte RV, Wu DC, Bigam D, Shapiro AM (2002). Effect of core pancreas temperature during cadaveric procurement on human islet isolation and functional viability. Transplantation.

[28] van Loo ES, Krikke C, Hofker HS, Berger SP, Leuvenink HG, Pol RA (2017). Outcome of pancreas transplantation from donation after circulatory death compared to donation after brain death. Pancreatology.

[29] Shahrestani S, Webster AC, Lam VW, Yuen L, Ryan B, Pleass HC (2017). Outcomes from pancreatic transplantation in donation after cardiac death: a systematic review and meta-analysis. Transplantation.

[30] Anderson PT, Aquil S, McLean K, McAlister VC, Sener A, Luke PP (2017). First Canadian experience with donation after cardiac death simultaneous pancreas and kidney transplants. Can J Surg.

[31] Kopp WH, Lam HD, Schaapherder AFM, Huurman VAL, van der Boog PJM, de Koning EJP, de Fijter JW, Baranski AG, Braat AE (2018). Pancreas transplantation with grafts from donors deceased after circulatory death: 5 years single-center experience. Transplantation.

[32] Fridell JA, Mangus RS, Thomas CM, Kubal CA, Powelson JA (2017). Donation after circulatory arrest in pancreas transplantation: a report of 10 cases. Transplant Proc.

[33] Mitchell T, Saba H, Laakman J, Parajuli N, MacMillan-Crow LA (2010). Role of mitochondrial-derived oxidants in renal tubular cell cold-storage injury. Free Radic Biol Med.

[34] Hosgood SA, Nicholson ML (2010). The role of perfluorocarbon in organ preservation. Transplantation.

[35] Branchereau J, Renaudin K, Kervella D, Bernadet S, Karam G, Blancho G, Cantarovich D (2018). Hypothermic pulsatile perfusion of human pancreas: preliminary technical feasibility study based on histology. Cryobiology.

[36] Barlow AD, Hosgood SA, Nicholson ML (2013). Current state of pancreas preservation and implications for DCD pancreas transplantation. Transplantation.

[37] Dholakia S, Royston E, Sharples EJ, Sankaran V, Ploeg RJ, Friend PJ (2018). Preserving and perfusing the allograft pancreas: past, present, and future. Transplant Rev (Orlando).

[38] Scott WE, Weegman BP, Ferrer-Fabrega J, Stein SA, Anazawa T, Kirchner VA (2010). Pancreas oxygen persufflation increases ATP levels as shown by nuclear magnetic resonance. Transplant Proc.

[39] Scott WE, O'Brien TD, Ferrer-Fabrega J, Avgoustiniatos ES, Weegman BP, Anazawa T (2010). Persufflation improves pancreas preservation when compared with the two-layer method. Transplant Proc.

[40] Caballero-Corbalan J, Eich T, Lundgren T, Foss A, Felldin M, Kallen R (2007). No beneficial effect of two-layer storage compared with UW-storage on human islet isolation and transplantation. Transplantation.

[41] Magnus R (1902). Die Thätigkeit des überlebenden Säugethierherzens bei Durchströmung mit Gasen. Arch Exp Path Parmakol.

[42] Fischer JH, Kuhn-Regnier F, Jeschkeit S, Switkowski R, Bardakcioglu O, Sobottke R (1998). Excellent recovery after prolonged heart storage by preservation with coronary oxygen persufflation: orthotopic pig heart transplantations after 14-hr storage. Transplantation.

[43] Flatmark A, Slaattelid O, Woxholt G (1975). Gaseous persufflation during machine perfusion of human kidneys before transplantation. Eur Surg Res.

[44] Rolles K, Foreman J, Pegg DE (1984). Preservation of ischemically injured canine kidneys by retrograde oxygen persufflation. Transplantation.

[45] Minor T, Isselhard W (1996). Synthesis of high energy phosphates during cold ischemic rat liver preservation with gaseous oxygen insufflation. Transplantation.

[46] Reddy MS, Carter N, Cunningham A, Shaw J, Talbot D (2014). Portal venous oxygen persufflation of the donation after cardiac death pancreas in a rat model is superior to static cold storage and hypothermic machine perfusion. Transpl Int.

[47] Kelly AC, Smith KE, Purvis WG, Min CG, Weber CS, Cooksey AM, Hasilo C, Paraskevas S, Suszynski TM, Weegman BP, Anderson MJ, Camacho LE, Harland RC, Loudovaris T, Jandova J, Molano DS, Price ND, Georgiev IG, Scott WE, Manas DMD, Shaw JAM, OʼGorman D, Kin T, McCarthy FM, Szot GL, Posselt AM, Stock PG, Karatzas T, Shapiro AMJ, Lynch RM, Limesand SW, Papas KK (2019). Oxygen perfusion (persufflation) of human pancreata enhances insulin secretion and attenuates islet proinflammatory signaling. Transplantation.

[48] Moers C, Pirenne J, Paul A, Ploeg RJ (2012). Machine preservation trial study group. Machine perfusion or cold storage in deceased-donor kidney transplantation. N Engl J Med.

[49] Westerkamp AC, Karimian N, Matton AP, Mahboub P, van Rijn R, Wiersema-Buist J (2016). Oxygenated hypothermic machine perfusion after static cold storage improves hepatobiliary function of extended criteria donor livers. Transplantation.

[50] Tersigni R, Toledo-Pereyra LH, Pinkham J, Najarian JS (1975). Pancreaticoduodenal preservation by hypothermic pulsatile perfusion for twenty-four hours. Ann Surg.

[51] Toledo-Pereyra LH, Valgee KD, Castellanos J, Chee M (1980). Hypothermic pulsatile perfusion: its use in the preservation of pancreases for 24 to 48 hours before islet cell transplantation. Arch Surg.

[52] Kenmochi T, Asano T, Nakagouri T, Enomoto K, Isono K, Horie H (1992). Prediction of viability of ischemically damaged canine pancreatic grafts by tissue flow rate with machine perfusion. Transplantation.

[53] Hamaoui K, Gowers S, Sandhu B, Vallant N, Cook T, Boutelle M, Casanova D, Papalois V (2018). Development of pancreatic machine perfusion: translational steps from porcine to human models. J Surg Res.

[54] Taylor MJ, Baicu S, Greene E, Vazquez A, Brassil J (2010). Islet isolation from juvenile porcine pancreas after 24-h hypothermic machine perfusion preservation. Cell Transplant.

[55] Taylor MJ, Baicu S, Leman B, Greene E, Vazquez A, Brassil J (2008). Twenty-four hour hypothermic machine perfusion preservation of porcine pancreas facilitates processing for islet isolation. Transplant Proc.

[56] Weegman BP, Taylor MJ, Baicu SC, Scott WE, Mueller KR, Kitzmann JD (2012). Hypothermic perfusion preservation of pancreas for islet grafts: validation using a split lobe porcine model. Cell Med.

[57] Karcz M, Cook HT, Sibbons P, Gray C, Dorling A, Papalois V (2010). An ex-vivo model for hypothermic pulsatile perfusion of porcine pancreata: hemodynamic and morphologic characteristics. Exp Clin Transplant.

[58] Leeser DB, Bingaman AW, Poliakova L, Shi Q, Gage F, Bartlett ST, Farney AC (2004). Pulsatile pump perfusion of pancreata before human islet cell isolation. Transplant Proc.

[59] Barlow AD, Hamed MO, Mallon DH, Brais RJ, Gribble FM, Scott MA, Howat WJ, Bradley JA, Bolton EM, Pettigrew GJ, Hosgood SA, Nicholson ML, Saeb-Parsy K (2015). Use of ex vivo normothermic perfusion for quality assessment of discarded human donor pancreases. Am J Transplant.

[60] op den Dries S, Karimian N, Sutton ME, Westerkamp AC, Nijsten MW, Gouw AS (2013). Ex vivo normothermic machine perfusion and viability testing of discarded human donor livers. Am J Transplant.

[61] Hosgood SA, Barlow AD, Hunter JP, Nicholson ML (2015). Ex vivo normothermic perfusion for quality assessment of marginal donor kidney transplants. Br J Surg.

[62] Kuan KG, Wee MN, Chung WY, Kumar R, Mees ST, Dennison A, Maddern G, Trochsler M (2017). A study of normothermic hemoperfusion of the porcine pancreas and kidney. Artif Organs.

